# Budesonide: A promising candidate therapeutic for early COVID-19

**DOI:** 10.1016/j.amsu.2021.102605

**Published:** 2021-08-02

**Authors:** Shailesh Kumar Patel, Rakesh Kumar, Jigyasa Rana, Aditya Agrawal, Alok Singh, Adesh Kumar

**Affiliations:** Department of Veterinary Pathology, College of Veterinary Science and Animal Husbandary, Bilaspur, CGKV, Chhattisgarh, India; Department of Veterinary Pathology, Dr. G.C. Negi College of Veterinary and Animal Sciences, CSK Himachal Pradesh Agricultural University, Palampur, Himanchal Pradesh, India; Department of Veterinary Anatomy, Faculty of Veterinary and Animal Sciences, RGSC, BHU, Barkachha, Mirzapur, Uttar Pradesh, India; Division of Animal Biochemistry, ICAR- Indian Veterinary Research Institute, Bareilly, Izatnagar, Uttar Pradesh, India; Division of Pathology, ICAR-Indian Veterinary Research Institute, Izatnagar, Uttar Pradesh, India; Veterinary Assistant Surgeon, Veterinary Hospital Aswar, Bhind, Madhya Pradesh, India

**Keywords:** Budesonide, COVID-19, Inhaled, Corticosteroid, Drug, Clinical trial

The severe acute respiratory syndrome coronavirus 2 (SARS-CoV-2) was emerged in China and challenged the global researchers for developing specific therapeutics that can halt the rapidly spreading ongoing pandemic. However, the development of a specific and effective therapeutic against SARS-CoV-2 may take several years and prove very costly. Therefore, many drugs were tested in order to repurpose them for COVID-19 and few of them received emergency use authorization also from global health agencies after completion of their clinical trials but a specific, safe, approved and effective treatment regimen is still awaited. In addition, the death toll and fear of the virus among global population is rising day by day in absence of specific drug, leading to severe psychological trauma.

The earlier studies reported that the onset of COVID-19 is usually mild and advances with time to severe disease attributed to the virus induced cytokine storm [1]. In this context, a potential window is available between mild and severe disease which can be intervened in order to prevent severe disease [2]. In contrast to this, most of the studies involved and targeted severe COVID-19 cases in search of a potential treatment regimen [3]. Very limited studies are available on the treatment strategies of early COVID-19 in order to prevent progression and clinical deterioration leading to death. In this context, an easily accessible effective COVID-19 treatment is urgently required for early COVID-19 patients with mild disease not requiring hospitalization. Hence, an open-label, parallel-group, phase 2, randomised controlled trial (Steroids in COVID-19; STOIC) of inhaled budesonide (NCT04416399) was performed in individuals with early COVID-19 in the community. In the STOIC trial 146 participants were randomly assigned to inhaled budsonide or usual care within 7 days of the onset of mild COVID-19 symptoms. The results of STOIC trial revealed that early administration of inhaled budesonide reduced the likelihood of needing urgent medical attention and reduced the time to recovery following early COVID-19 [4]. In addition, a multicenter, open-label, multi-arm PRINCIPLE trial (ISRCTN86534580) also enrolled people over the age of 65 or people over the age of 50 with a history of comorbidities and the interim analysis of the phase 3 PRINCIPLE clinical trial revealed promising results of budesonide in treating patients with mild COVID-19 infection. The interim results of PRINCIPLE trial reported that the patients taking inhaled budesonide had a faster COVID-19 recovery time by 3 days than patients who received only usual care along with lower hospitalizations in the budesonide group than the usual care group [5]. Therefore, the UK government said that budesonide was “not currently being recommended as standard of care but can be considered (off label) on a case-by-case basis for symptomatic covid-19 positive patients aged 65 and over, or aged 50 or over with co-morbidities” [6].

Budesonide ([RS]-1β, 16α 17, 21-tetrahydroxypregna-1, 4-diene-3, 20-dione cyclic 16, 17-acetal with butyraldehyde) is a non-halogenated corticosteroid that exhibits predominantly glucocorticoid activity along with a weak mineralocorticoid activity (Fig. 1) [7]. The budesonide is a potent topical anti-inflammatory agent which binds and activates glucocorticoid receptors (GR) present in the cytoplasm of effector cell, which allows the translocation of this budesonide-GR complex in the nucleus. Moreover, budesonide prevents the expression of pro-inflammatory genes in the nucleus and increase the expression of anti-inflammatory genes, which results in the reduction in formation of the inflammatory cytokines such as ILs and TNF (Fig. 2) [8]. Additionally, budesonide also inhibits the eosinophil activation by increasing apoptosis and suppresses the activation of various inflammatory cells such as neutrophils, mast cells, macrophages, T-lymphocytes, and dendritic cells [9]. The effect of overall inhibition of ILs and TNF produced by budesonide leads to reduced airway inflammation and hyperreactivity resulting into inhibition of the bronchospasm and subsequently wheezing and coughing [8].

**Fig. 1:**
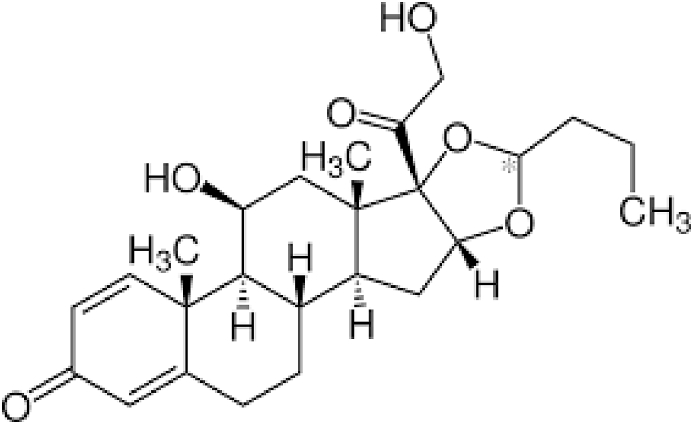
Chemical structure of budesonide.

**Fig. 2:**
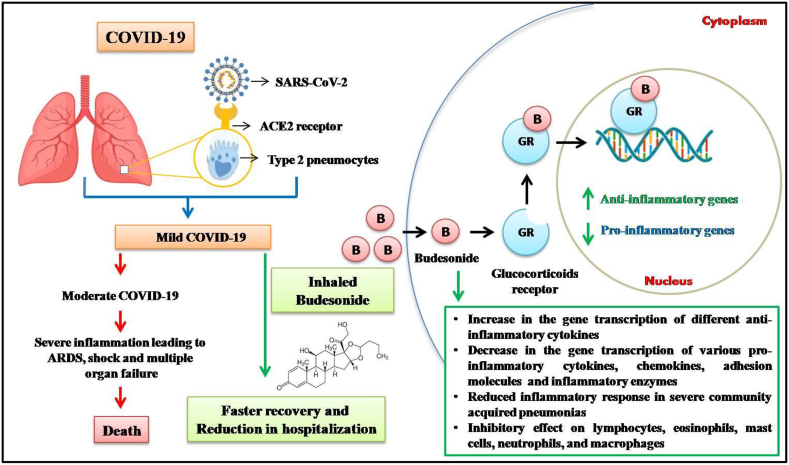
Mechanism of action and an overview of budesonide in COVID-19 patients.

Several randomized trials have already demonstrated the clinical benefits of systemic corticosteroids such as dexamethasone in the treatment of critically ill people hospitalized with COVID-19 [10]. A total of five clinical trials involving budesonide for the treatment of COVID-19 are currently evaluating the efficacy of budesonide in COVID-19 patients (Table 1). However, the inhaled glucocorticoids are mainly used in the patients of asthma and COPD to reduce exacerbations which may be due to a viral cause too [11], but the findings of the STOIC and PRINCIPLE trial suggest that early treatment by the inhaled corticosteroids such as budesonide would be effective and relevant for clinical practice in COVID-19. In addition, The In-vitro studies reported that inhaled glucocorticoids reduce the SARS-CoV-2 replication in airway epithelial cells [12] and downregulates the expression of ACE2 and TMPRSS2 genes critical for cell entry of the virus [13] further supports the use of budesonide in COVID-19 cases.

**Table 1:** Clinical trials evaluating the efficacy of budesonide in COVID-19 patients (www.clinicaltrials.gov).

S. No.	NCT Number	Title	Phase	Interventions	Populations
1	NCT04331470	Evaluation of Efficacy of Levamisole and Formoterol +Budesonide in Treatment of COVID-19	Phase 2 Phase 3	•Drug: Levamisole Pill + Budesonide +Formoterol inhaler •Drug: Lopinavir/Ritonavir + hydoxychloroquine	Enrollment:30 Age: 15-100 Years (Child, Adult, Older Adult) Sex: All
2	NCT04361474	Trial Evaluating the Efficacy of Local Budesonide Therapy in the Management of Hyposmia in COVID-19 Patients Without Signs of Severity (COVIDORL)	Phase 3	•Drug: Budesonide Nasal •Other: Physiological serum	Enrollment:120 Age: 18 Years and older (Adult, Older Adult) Sex: All
3	NCT04416399	STerOids in COVID-19 Study (STOIC)	Phase 2	•Drug: Budesonide dry powder inhaler	Enrollment:146 Age: 18 Years and older (Adult, Older Adult) Sex: All
4	NCT04355637	Inhaled Corticosteroid Treatment of COVID19 Patients With Pneumonia	Phase 4	•Drug: Inhaled budesonide	Enrollment:300 Age:18 Years to 79 Years (Adult, Older Adult) Sex: All
5	NCT04331054	Protective Role of Inhaled Steroids for Covid-19 Infection (INHASCO)	Phase 3	•Drug: 2: Usual practice + SYMBICORT RAPIHALER •Other: 1: Usual practice	Enrollment:436 Age: 18 to 75 Years (Adult, Older Adult) Sex: All

Although, clinical benefits of budesonide in COVID-19 patients is not well established but speeding recovery and reduction in hospitalizations of early COVID-19 cases followed by inhaled budesonide can reduce current pressure on health care systems which is already collapsed in most of the developing countries. Inhaled budesonide is a simple, safe, very well studied, widely available, and inexpensive corticosteroid which may prove crucial for mild COVID-19 cases. Additionally, budesonide could give healthcare workers more options in treating COVID-19 patients, especially as it is readily available in most of the primary healthcare settings and is listed as Essential Medicine in the World Health Organization's List of Essential Medicines. Moreover, the budesonide can be used with ease even in comorbid, unwell, and potentially frail older patients.

In conclusion, the fate of budesonide depend on the results of ongoing clinical trials but till date it appears as a promising candidate therapeutic for mild COVID-19 cases and may prove crucial in halting the disease in early form by shortening the recovery time and reducing the need of hospitalization. Moreover, studies targeting the budesonide as therapeutic agent in early COVID-19 are highly warranted in order to include it in early clinical management of COVID-19. However, before including budesonide in the approved treatment regimen and widespread use against SARS-CoV-2, its efficacy and safety must be established by using suitable animal model and cell lines.

## Author contributions

All the authors substantially contributed to the conception, design, analysis, and interpretation of data, checking, and approving the final version of the manuscript, and agree to be accountable for its contents.

## Data statement

Data sharing is not applicable to this article as no new data were created or analysed in this study.

## Provenance and peer review

Not commissioned, internally peer-reviewed.

## Sources of funding

None.

## Ethical approval

Not applicable.

## Trial registry number

1. Name of the registry: Not applicable
2. Unique Identifying number or registration ID: Not applicable
3. Hyperlink to your specific registration (must be publicly accessible and will be checked): Not applicable

## Guarantor

Shailesh Kumar Patel and Rakesh Kumar accept the full responsibility for the work and controlled the decision to publish.

## Declaration of competing interest

All authors declare that there exist no commercial or financial relationships that could, in any way, lead to a potential conflict of interest.
